# Experimental Evaluation of Polyphenylsulfone (PPSF) Powders as Fire-Retardant Materials for Processing by Selective Laser Sintering

**DOI:** 10.3390/polym13162704

**Published:** 2021-08-13

**Authors:** Yifan Lv, Wayne Thomas, Rodger Chalk, Andrew Hewitt, Sarat Singamneni

**Affiliations:** 1 Additive Manufacturing Research Centre, Auckland University of Technology, WS116, 34 Saint Paul Street, Auckland CBD, Auckland 1010, New Zealand; yifan.lv@aut.ac.nz; 2 Air New Zealand, 5 Geoffrey Roberts Road, Mangere, Auckland 2022, New Zealand; wayne.thomas@airnz.co.nz (W.T.); rodger.chalk@airnz.co.nz (R.C.); andrew.hewitt@airnz.co.nz (A.H.)

**Keywords:** polyphenylsulfone, PPSF, fire-resistant, additive manufacturing, aircraft interior, selective laser sintering

## Abstract

Additive manufacturing has progressed rapidly, and the unique attributes of the layer-wise material consolidation are attracting ever increasing application potentials in critical sectors such as medical and aerospace industries. A lack of materials options has been the main bottleneck for the much wider uptake of these promising new technologies. Inventing new material alternatives has been central to most of the research attention in additive manufacturing in recent times. The current research is focused on evaluating the polyphenylsulfone polymer powders for the first time as fire-resistant candidate materials for processing by selective laser sintering, the most promising additive processing method for polymeric material systems. Experimental evaluations were undertaken based on a selective laser sintering test bed. Single layer and multi-layer samples were produced for microstructural and mechanical characterisations. The microstructural evaluations and the mechanical property results indicate sufficient intra- and inter-layer consolidation together with reasonable tensile property responses. The lower viscosity and thermal conductivity characteristics rendered lower tensile strengths, which will require some further attention in the future, for better consolidation and mechanical properties.

## 1. Introduction

Considering their highly inflammable nature and toxicity, fire-safe qualities are mandatory for polymer materials used in aero-space applications, which are normally achieved by chemical or physical treatments [1,2,3,4,5]. The additives often may adversely affect the environment or the properties of the fire-resistant polymer [6,7] but intrinsically fire-resistant options such as poly ether ketone (PEEK) have proved to be effective [8]. Several flame-retardant polymers are effectively in use for aircraft interior parts, confirming to the FAR 25.855 standards [9,10,11]. However, the-e has been a never-ending urge to identify new materials and processes for more efficient building of fire-retardant interior components of aircrafts [12].

Injection moulding [13], hand lay-up, spray-up, compression moulding, filament winding, pultrusion, resin transfer moulding, vacuum-assisted resin transfer moulding, infusion, and continuous panel processing [1] are common methods traditionally used for making fire-resistant polymer parts for the aircraft interior. While these are well-developed manufacturing solutions, the production lead times are often high and the supply-chains quite intricate. Additive manufacturing methods have recently evolved from the erstwhile rapid prototyping stages, offering potential new material processing solutions with better freedom to achieve more complex and optimum design forms. In particular, with the possibility to simplify the supply-chain and inventory constraints, these new methods are attracting significant attention in the aircraft manufacturing and maintenance tasks [14].

Two of the most common AM techniques for processing polymers are selective laser sintering (SLS) and fused deposition modeling (FDM) based on powder bed fusion and material extrusion techniques, respectively. It is well known that the components processed by SLS or by FDM show properties, which heavily depend on the building orientation and processing parameters. In SLS, the main parameters to be optimised are the laser power, the laser scanning pattern, and speed and the hatch distance [15]. For FDM technique, on the other hand, the extrusion speed, deposition temperature of filament, infill percentage, and raster angle and strategies play critical roles [16]. In both techniques, the parts produced can show different mechanical properties depending on those processing orientation and parameters, as they affect the intra- and inter-layer conditions. This is due to the very nature of additive technologies, producing components in a point-wise material consolidation manner. The advantages and disadvantages, and a general comparison between the two techniques are concluded in [15,17,18]. Summarising, FDM is relatively cheap, allows multi-colour solutions, bigger build sizes, and low post-processing costs. On the other hand, SLS needs no support structures, gives better surface quality and part definitions, allowing to produce movable joints with high resolution and close tolerances. However, FDM is inferior to SLS in terms of part quality, anisotropy of the material consolidation, stair-step problems, higher porosity levels, and the need for support structures. The main drawback with SLS is the limited materials options currently available and highly proprietary nature of the materials that are already in use. Further, SLS parts generally have superior mechanical strength and less anisotropy [17]. For example, with Poly-Ether-Ether-Ketone (PEEK), the tensile strengths are 40 to 55 MPa [19,20], and 80 to 90MPa [21] when processed by FDM and SLS, respectively.

Despite the numerous benefits additive manufacturing can bring to the polymer-based manufacturing of aircraft parts, the progress so far has been limited, and mostly confined to the fused deposition modelling (FDM) methods [22]. Proprietary materials and the black-box-type commercial systems have rendered obstacles for the wide range exploration of different materials and processes in applying the additive methods to the needs of the aircraft industry [23]. In particular, the stairstep effects, fibre discontinuity, and the meso-structural limitations [16] typical of FDM render inherent weaknesses in the consolidated material structures. Selective laser sintering (SLS), on the other hand, is a powder bed fusion process for polymers and the point-by-point laser induced energy allows to achieve a better material consolidation mechanics through controlled inter-particle coalescence [24].

There is also a dearth of materials options for processing aircraft parts using fire-retardant polymeric materials. In particular, the laser sintering route lacks a variety of materials options to choose from [25]. Polyphenylsulfone (PPSF or PPSU) is an amorphous thermoplastic, which is also intrinsically flame retardant, with a decent mechanical strength, and can be a potential competitor to PEEK and Ultem. Polyphenylsulfone is mostly researched for membrane material solutions [26], while some studies show that it can also be processed by injection moulding [13]. The tensile strength of polyphenylsulfone is around 70 MPa. There have been some reports, indicating that it can be processed by FDM [27], achieving mechanical properties comparable with Ultem and polycarbonate. However, there has been no evidence of this material being researched for processing by selective laser sintering. The current paper addresses this research gap through experimental investigations, leading to the understanding of how polyphenylsulfone powders respond to consolidation by laser sintering with varying energy inputs. The results indicate the material to be responding positively to consolidation by the continuously moving laser energy input, though the time and temperature conditions lead to specific challenges in controlling the resulting meso-structures and the mechanical properties.

## 2. Material and Methods

**Figure 1:**
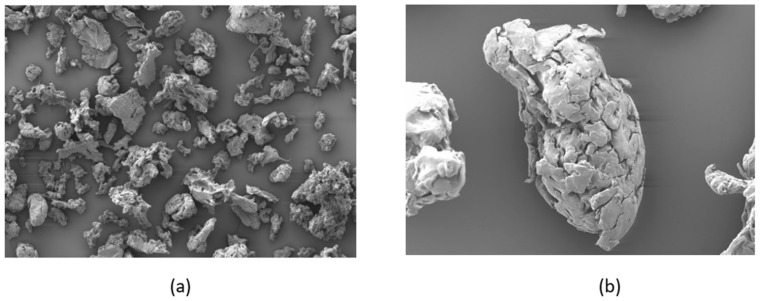
Scanning electron microscope (SEM) images of PPSF powder particles, (**a**): 100× zoom, (**b**): 500× zoom.

Polyphenylsulfone (PPSF/PPSU) is an amorphous polymer with a repeating unit of molecular form (-C_6_H_4_-4-SO_2_C_6_H_4_-4-OC_6_H_4_-4-C_6_H_4_-4-O-)_n_. The molecular weight is 1600 g mol^−1^. The powder was specially ordered from Galaxy Chemical Technology Co Ltd., Shenzhen, Guangdong, China. This material is not available in powder form in the commercial sales market. Since this was the first time the powder was ordered, Galaxy Chemical Technology used the cryo-grinding method to mechanically prepare the powder samples. As a result, the particle morphologies were too complex and also quite varied. However, the average powder particle size is around 42 microns. The powder samples were coated with platinum and examined under the Hitachi SU-70 Scanning electron microscope (SEM) system, and the scanning voltage was set as 15 KV to avoid charging effects. SEM images of the PPSF powder particles shown in Figure 1 clearly indicate the irregular forms and variations in sizes. These irregular shapes of the particles caused by poor cryogenic grinding will hinder the flowability of the powder on the build platform of the laser sintering test bed. In addition, the varying sizes can cause problems to the consolidation of layers based on the heat input from a fast-moving laser energy [28,29,30,31,32,33,34,35].

**Figure 2:**
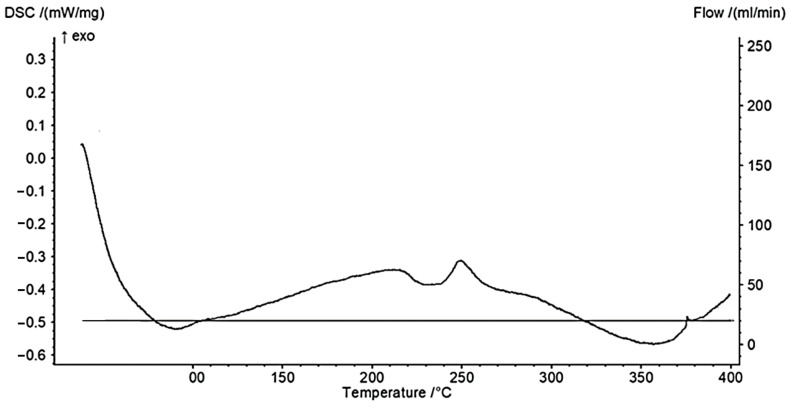
Differential scanning calorimetry (DSC) results based on the PPSF powder.

Differential scanning calorimetry (DSC) is used to measure the heat into or out of the test polymer powder, generating a thermal profile that can be used for establishing the sintering window of these powder materials, before undertaking the laser sintering trials. The DSC system NETZSCH STA 449F5 STA449F5A-0062-M was used to identify the thermal profile of the PPSF powders and to establish the promising ranges of the critical process parameters. A mass of 4.9 mg of PPSF powder was heated from 20 °C to 400 °C with 10.0 °C/min heating gradient during the heating cycle. The DSC curve obtained is shown in Figure 2. Based on the exothermic reaction up as the convention, the small step observed at around 220 °C was taken as the glass transition temperature. The information from the DSC graph was used to establish the critical process parameters for the laser sintering trials.

All the laser sintering trials were performed on a homemade selective laser sintering test bed available at the Additive Manufacturing Research Centre of the Auckland university of Technology. The experimental setup constituted of a 60 W CO_2_ laser with sufficient control on the pulse rate and the raster scanning on the powder bed. The powder bed was also tailor-made for semi-automatic dispersal of powders in small quantities for experimental study of the feasibility of laser sintering single and multi-layer specimens with varying process conditions. The complete details of the experimental setup may be obtained from the reference by Velu et al. [28].

The theoretical energy density (ED) necessary to sinter a given quantity of the PPSF powder was calculated based on the procedure used by Berretta et al. [29]. Laser powder (p), scan speed (v), and laser beam diameter (D) are the process parameters. The powder bed temperature ($T_{b}$) was set at 200 °C, which is just below the glass transition temperature of PPSF. Single-layer samples were made with varying process parameters in order to evaluate the particle coalescence. Based on the literature, the enthalpy of relaxation ($h_{r}$) of PPSF is around 0.73 J g^−1^ [30], which is very small due to the amorphous nature of the material. The bulk density (Q) and the packing factor (φ) were estimated as 0.350 g cm^−3^ and 0.8, respectively. The layer thickness (z) is 0.20 mm. $T_{o}$ is the onset temperature during laser sintering. The specific heat capacity, ($C_{pb}$) of PPSF varies with temperature and at the powder bed temperature is around 0.44 cal g^−1^ °C^−1^. The specific heat capacity at the onset sintering temperature ($C_{po}$) is assumed based on a linear approximation. Based on this assumption, Equation (1) can be used to evaluate the theoretical energy required for sintering. Equation (2) is the theoretical energy density for single layer sintering, which can be used to derive the expression for the sintering energy density for materials with specific heat capacity varying as a function of temperature. Based on a linear approximation within the sintering temperature window, the specific heat capacity can be expressed as in Equation (4). Combining Equations (1)–(4), the semi-empirical Equation (5) can be derived, which can be used to calculate the experimental energy density:

(1) $$Energy\;required\;for\;sintering=[C_{p}\left(T_{o}-T_{b}\right)+h_{r}]Q\varphi$$

(2) ED= Energy required for sintering ×z

(3) $$ED=\left\{\int_{T_{b}}^{T_{o}}[\frac{dC_{p}}{dT}\left(T_{o}-T_{b}\right)+h_{r}]Q\varphi \right\}z$$

(4) $$C_{p}=\frac{\left(T-T_{o}\right)\left(C_{pb}-C_{po}\right)}{\left(T_{b}-T_{o}\right)}+C_{po}$$

(5) $$ED=\left\{h_{r}Q\varphi +Q\varphi \int_{T_{b}}^{T_{o}}\left[\frac{\left(T-T_{o}\right)\left(C_{pb}-C_{po}\right)}{\left(T_{b}-T_{o}\right)}+C_{po}\right]dT\right\}z$$

Considering that the polymer particles are irregular, the size of the particle and the variation of the viscosity with temperature will affect the sintering time, which in turn will influence the inter-particle coalescence during sintering [31]. According to the Frenkel’s model, the higher the product of the viscosity and the particle radius, the longer the sintering time required [31]. The geometrical parameters were measured based on the SEM images, which indicate the original radius, sintered radius, and distance from the centre to the sintering plane of two adjacent particles. Additionally, other properties such as surface tension and viscosity were related to the time (t) of sintering as well, using the information reported by Sedlacek et al. and Mohan [32,33]. The classical sintering equation proposed by Frankel is only suitable for a 2D simplification. The modified Frenkel model proposed by Sun et al. [34] can be used for a 3D situation, which allowed to establish the working energy density of the current powders as discussed next.

**Figure 3:**
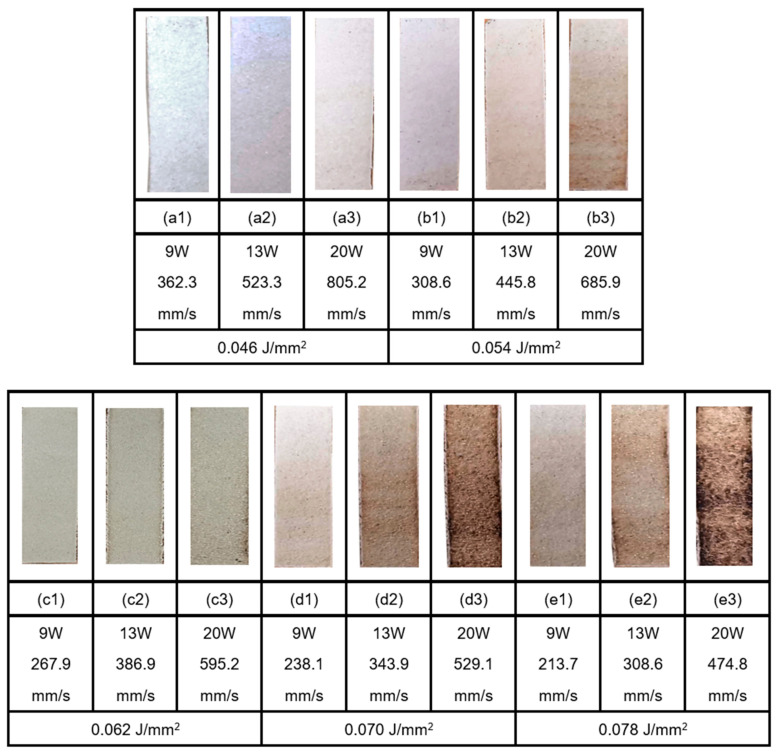
Single layer samples of PPSF produced by SLS; the energy density of 0.046 J/mm^2^, 0.054 J/mm^2^, 0.062 J/mm^2^, 0.070 J/mm^2^, and 0.078 J/mm^2^ correspond with group (**a**–**e**), respectively. Laser power of 9 W, 13 W, and 20 W are used for the first, second, and third sample in the group, respectively, with varied scanning speed. All samples are of 30 × 100 mm size.

**Figure 4:**
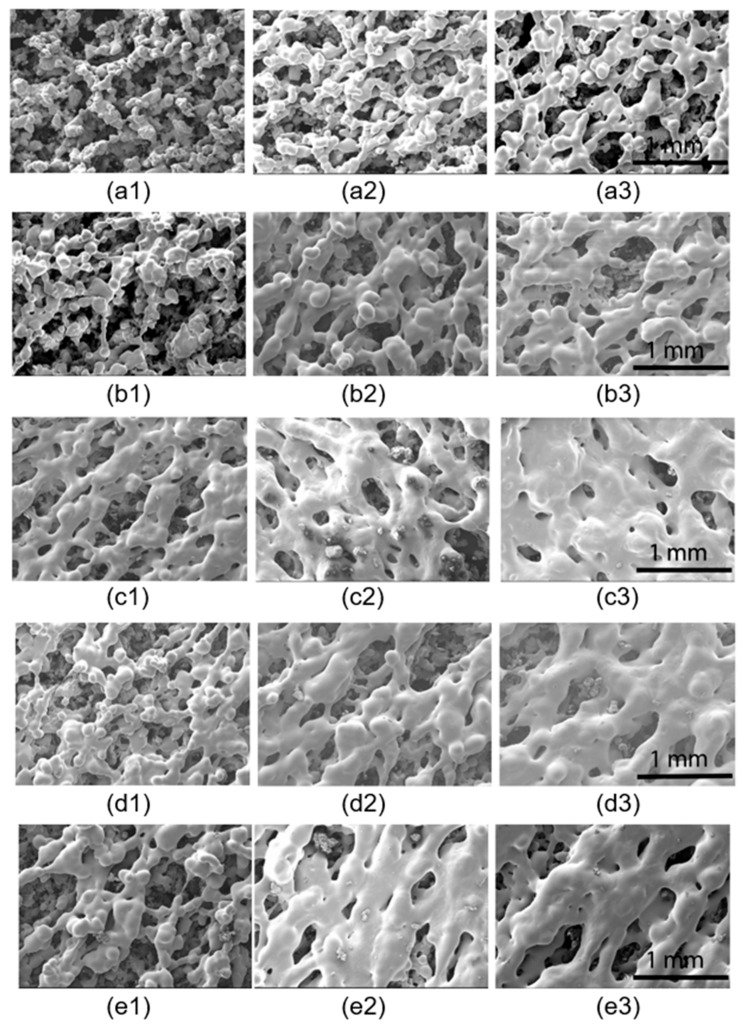
SEM images of single layer samples of PPSF produced by SLS. The energy density of 0.046 J/mm^2^, 0.054 J/mm^2^, 0.062 J/mm^2^, 0.070 J/mm^2^, and 0.078 J/mm^2^ correspond with group (**a**–**e**), respectively. Laser power of 9 W, 13 W, and 20 W are used for the first, second, and third sample in the group, respectively, with varied scanning speed.

Once the sintering across a single layer was thus established, a series of specimens were produced with the energy densities varied at 0.046, 0.054, 0.062, 0.07, and 0.078 J/mm^2^; the images of the printed samples are presented in Figure 3. Three levels of laser power settings were used for each energy density—9 W, 13 W, and 20 W—together with the corresponding scan speeds, as stated in Figure 3. A total of 45 single layer samples were produced considering three single layer sample replicas for each process parameter combination. The photographs of the best of the three printed samples with each process parameter combination are shown in Figure 3. These samples are subsequently examined using SEM and the images obtained are presented in Figure 4. Single layer or the first layer is sintered on a solid substrate and not on loose powder. The solid substrate is often made with one material or a material with similar characteristics. Hence, there is relatively lesser variation from single to multi-layers. However, thermal stability issues arise in multi-layer samples as the heat from the substrate cannot easily cross the multi-layers. The external heat source in the form of a heating lamp will compensate and keep the conditions almost similar.

Porosity of the printed samples was established based on the analysis of the SEM images of the single layer sintered samples using the ImageJ software, which is done by the same authors in another study [35]. Three SEM images were taken using samples selected from the single layer samples printed with each of the process parameter combinations. All the images are then used to process by the ImageJ software to analyse the porosity levels. The total specimen areas and number and average sizes of the lack of fusion areas, established from the images processed by ImageJ software, were used to calculate the percent porosity levels. Samples for tensile testing were printed as per the standard ASTM D638. All the tensile tests were performed using the Tinius Olsen H50KS. A S-Beam load cell was used for the test, loading at the rate one millimetre per minute. Each tensile test specimen was printed processing five layers of the PPSF powder, with the process conditions, as discussed in the results section.

## 3. Results and Discussions

From the calculations based on the theoretical modelling explained in the previous section, it was established that the sintering rate reaches 2.277 × 10^−4^ μm/s, when the onset temperature to be 360 °C. This is theoretically sufficient to achieve sufficient consolidation through selective laser sintering. Based on this number, the theoretical energy density necessary to achieve sufficient consolidation was established to be 0.022 J/mm^2^ using Equation (5). It is common for the theoretical energy density to be lower than the actual energy density required. Starting with this as the first step, the actual energy density necessary was established based on trial-and-error methods. After a few iterations, the first successful single layer specimen with significant inter-particle coalescence was achieved with an energy density of 0.046 J/mm^2^, which is twice that of the theoretically estimated energy density. While there are differences in the real thermal conditions and the theoretical estimates, there is also the problem of heat loss from the powder bed, which is not strictly considered in the theoretical calculations

The images of the single layer samples presented in Figure 3 indicate very smooth surface textures and continuous formation of the layers based on powder consolidation achieved by the moving laser beam. There is a physical variation in the form of gradually changing colour from the first set to the last. Evidently, as the energy density increases, the polymer composite is gradually degraded and gets darker possibly due to decomposition and charring. However, a physical examination also revealed that the strength of the sintered samples increases with increasing energy densities, as the excess energy was able to consolidate the particles better. The samples became brittle and charred when the energy density is increased to 0.070 J/mm^2^. Based on a compromise between the physically observed strength and consolidation against the degradation, the best energy density for this polymer composite was estimated to be at around 0.062 J/mm^2^.

According to a study by Ramgobin et al. under thermo-oxidative atmosphere, the onset of the degradation temperature of PPSF is around 500 °C. With enough activation energy, a two-step decomposition will be activated, first the cleavage of phenyl–sulfone (Ph–SO2) linkages, and then the cleavage of Phenyl–Oxygen or Phenyl–Phenyl bonds. Through thermogravimetric analysis, it was shown that the residual mass of PPSF at 538 °C was at 95 wt% and as low as 40 wt% at 800 °C. This indicates that the loss of material with PPSF is insignificant during thermal degradation. However, charring is probably due to the residual elements of the products of pyrolysis of PPSF [36]. It was also stated that, when PPSU was heated under nitrogen, the degradation temperature is at 600 °C. It may be inferred that a N_2_ atmosphere in the build chamber would allow to avoid the decomposition and charring of the PPSF powders during selective laser sintering. Arnold et al. suggested that photothermal degradation of polymers caused by laser light may lead to ablation and loss of materials [37]. The ablation responses were correlated to the laser velocity and pulse rates. In the present case, the discolouration of the sintered samples could be due to the higher scan speeds used with the higher laser power settings.

Evidence of discolouration and curling of the specimens may also be noted based on the images of the sintered specimens shown in Figure 3. The discolouration of the edges could be due to the excessive heating that takes place at the end of each raster scan line. The can strategy is zig-zag lines across the width of the specimens. This means the laser will instantaneously stop at the end of each raster path, while the energy is flowing into the specimen at the same rate. This will lead to flow of excessive energy and the consequent discolouration on the longer edges of the specimens, as observed. Evidently there is no such discolouration on the top and the bottom edges of the samples, which is because of the fact that the laser scan lines are oriented parallel to these edges.

The images of the sintered specimens in Figure 3 also show indications of curling of the specimens over the edges. Apparently, the curling phenomenon is more prominent in the cases of the specimens produced at higher power and higher scan velocity settings within the ranges of each energy density, though to a lesser extent in the lower energy density cases. Curling takes place firstly due to lack of sticking of the sintered layer to the base plate. This can be easily fixed by suitably preparing the surface conditions. The uneven heat dispersion in the sintered layer also could cause the curling problem. The central areas are relatively at higher temperature compared to the edges, which may lead to contraction at the edge and consequent curling of the specimens. It is also possible that the excessive heating due to the instantaneous stopping and reversal of the laser beam at the end of each of the scan strokes could lead to embrittlement and loss of elastic nature at the edges, which can cause the curling effects. All these aspects can be controlled by adjusting the process conditions. The surfaces can be better prepared to promote more stronger sticking and the laser energy input can be adjusted to reduce to the necessary extent, as the beam gets closer to the ends of the raster scan lines.

Scanning electron microscopy images taken based on the single layer sintered samples shown in Figure 3 are presented in the same order in Figure 4. The energy density levels increase moving down the rows of images presented. Marked differences may be observed in the inter particle and intra-layer coalescence as the energy density is increased. Both inter-particle and intra layer consolidation is scarce in samples sintered with the lowest energy density 0.046 J/mm^2^, as evident from the images of Figure 4(a1–a3). The inter particle coalescence improved significantly showing considerable evidence of the formation of the consolidated polymer strands along the laser scan lines at the energy density 0.054 J/mm^2^, as may be seen in Figure 4(b1–b3). However, the inter-strand coalescence is still limited, and the layer formation is significantly restricted in terms of the resulting meso-structures, indicating a lack of energy to achieve sufficient powder consolidation. Both inter-particle and inter-strand coalescence improved with still higher energy densities above 0.062 J/mm^2^. Considering the overall dispersion of the consolidated materials and lack of fusion cavities, the energy density at 0.062 J/mm^2^ appears to be the optimum setting for effective laser sintering of this powder polymer.

Significant improvements in consolidation may also be visualised moving from the left image to the right, along any row of Figure 4. Though the energy density is the same for all the samples in a given row, the process parameter combination changes from low power and low velocity to high power and high velocity from the left to the right. Evidently, the powder consolidates better with a high power and high velocity setting for any given energy density level. This is often the case with many polymers as the power and velocity settings interact even at the same energy density levels. The high power and high velocity setting are preferred if the viscosity and thermal conductivity properties are lower. The higher power allows to heat the strands more. The lower viscosity leads to increased plasticisation and running of the plasticised strand, but the high velocity of scanning brings the laser back quickly along the next strand, allowing the softened adjacent polymer strands to fuse better. With polymers having lower thermal conductivities, the higher scan velocity helps to quickly move around and revisit the common points along adjacent strands and keep the thermal conditions elevated to the necessary levels promoting inter-strand coalescence [28,35].

Based on these observations, the best layer consolidation corresponds to Figure 4(c3,d3). In both cases, the laser power is the highest of the range used, at 20 W, with the laser scan velocity also at the highest levels corresponding to the energy density values used, 0.062 J/mm^2^ and 0.070 J/mm^2^, respectively. However, as the energy density goes beyond 0.062 J/mm^2^, the samples began to discolour significantly at the higher power setting 20 W. Considering both consolidation and deterioration responses, the energy density level at 0.062 J/mm^2^ with the higher power and velocity settings again appears to be the best possible process parameter conditions for the laser sintering of polyphenysulfone polymer powders.

**Figure 5:**
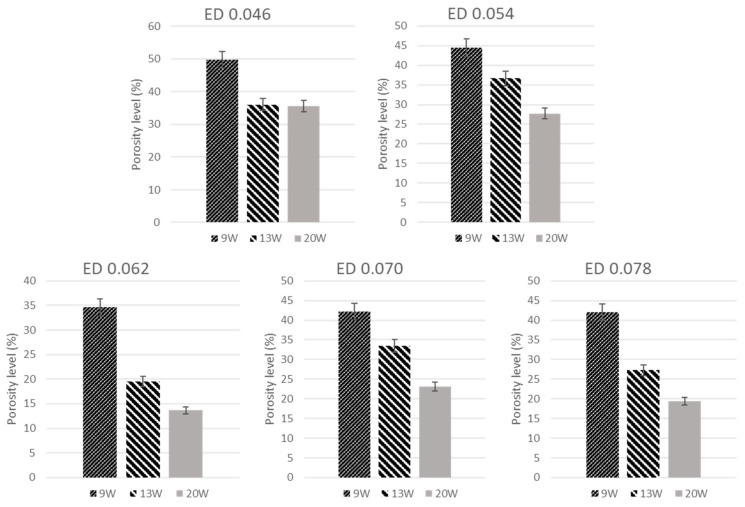
Porosity levels results of each processing parameter.

The results of the porosity analysis are presented as the bar charts with the percent porosity levels plotted against the laser power for each energy density, as depicted in Figure 5. It may be consistently observed that the porosity level decreases with increasing laser power, at any given laser energy density level. This is consistent with the observations made based on the examinations of the physical samples and SEM images, as discussed earlier. The lowest porosity level is based on the sample produced with laser power 20 W and energy density 0.062 J/mm^2^, which is in complete agreement with the inferences drawn from the other observations. It may also be noted that the porosity levels increase as the energy density level is increased to 0.070 J/mm^2^. There may be excessive heating happening when the energy density used is beyond the threshold value suitable for the polymer material investigated. The slightly reduced porosity levels at 0.078 J/mm^2^ could be misleading as the sintered layer appears to be overheated, decomposed, and charred and possibly blocking the meso-structural details. The porosity results corresponding to the energy density and laser power setting combinations 0.062 J/mm^2^ and 13 W, 0.062 J/mm^2^ and 20 W, and 0.078 J/mm^2^ and 20 W are considered to be the most optimum in terms of achieving the least porosity or the best sintered layer corresponding to the shortest bar heights in Figure 5.

**Figure 6:**
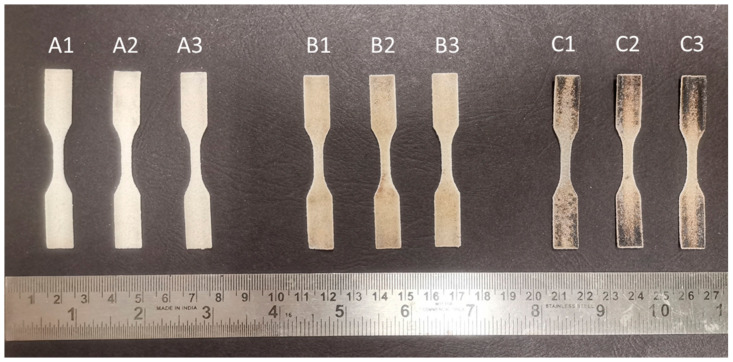
Multi-layered tensile test specimens of PPSF with three sets of laser power, scan speed and energy density combinations, which referring to Table 1.

Tensile test specimens were produced with the three process parameter combinations identified as the optimum settings from the porosity analysis. The process parameter settings and the tensile testing responses are listed in Table 1. Each tensile test specimen is printed as per the ASTM standards mentioned using five layers sintered one upon the other. The images of the printed test specimens are presented in Figure 6. Consistent dimensional and structural qualities could be observed in all the multi-layer tensile test specimens printed. There is some evidence of discolouration as may be noted with the high energy density and high-power samples in Figure 6(C1–C3). From a physical point of view, the specimens produced with 0.062 J/mm^2^ and 20 W corresponding to Figure 6(B1–B3) are the best possible outcomes based on selective laser sintering of the current polymer powders.

Comparing the tensile test results presented in Table 1, the first batch corresponding to the A-series scored the least in terms of the ultimate tensile strength, averaging at around 2.252 MPa. Both the B- and C- series samples are far better, averaging at 7.237 MPa and 8.652 MPa, respectively. This drastic loss of strength at the lower power setting 13 W is clearly indicative of the significant role of laser power in controlling the consolidation rates in the sintered polymer layers. The minimum threshold power is 20 W for the scan velocity ranges used in the current experimental work with laser sintering of polyphenylsulfone powders. The C-series produced at the highest energy density exhibit better ultimate tensile strengths than the C-series. However, there is discolouration and deterioration of the polymer, as evident from the images of the physical samples in Figure 6(C1–C3). Considering both ultimate tensile strength and the polymer degradation aspects, the B-series, corresponding to laser energy density 0.062 J/mm^2^ and power 20 W appear to be the best specimens produced within the range of factors used in the current experimental investigation. This is also in accordance with the observations made from the physical examination and SEM images and porosity analysis of the single layer samples. The experimental results show a clear trend and relationship between single layer porosity and multilayer properties. Thus, the single layer porosity can be correlated to the multilayer porosity and strength. The reported mechanical strength of PPSF via using fused deposition modelled techniques can achieve roughly 55 MPa [16]. However, the ultimate tensile strength results obtained from the laser sintered samples are much lower compared to the fused deposition modelled counter parts, as reported by Afrose et al. [38]. It may be pertinent to point out that the current results are only based on initial investigations targeted at evaluating the suitability of the polymer for processing by selective laser sintering. Form that viewpoint, the consolidation mechanisms observed clearly prove that polyphenylsulfone powders can be successfully processed by selective laser sintering. Further improvements in tensile properties can be achieved by a more scientific design of experiments considering all the critical process parameters. A careful consideration should be given to achieve uniform powder particle sizes and also closely controlling the atmosphere and temperature conditions inside the build chamber. A careful examination of the fractured surfaces of the tensile specimens clearly indicated brittle fracture modes. The results are satisfactory in terms of proving the PPSF powders for consolidation by laser sintering. However, the SLS test bed used is a make-shift laboratory experimental system. The mechanical properties will be much improved if a commercial system is used at later stages, where commercial applications are targeted.

## 4. Conclusions

Polyphenylsulfone powders are investigated for processing by selective laser sintering, based on experimental investigations with varying laser process parameter settings. Overall, the polymer powders responded well for the laser sintering process. Single and multilayer sintered samples were evaluated based on physical, meso-structural, porosity, and mechanical property examinations. Upon careful consideration of the results, the energy density level 0.062 J/mm^2^, achieved with laser power 20 W and scan speed 595.0 mm/s settings, were identified to be the most optimum process conditions. The best average ultimate tensile strength achieved in the multi-layer sintered samples was at around 8.652 MPa.

- Application of the Frenkel theory of sintering in a modified form resulted in an optimum ED of 0.062 J/mm^2^ for laser sintering PPSF powders.
- Experimental observations allowed to establish the best process conditions for single-layer sintering as ED 0.062 J/mm^2^, obtained with laser power 20 W and scan speed 595.0 mm/s.
- The consolidation mechanics varied with both geometry and the number of layers printed and for multilayer printing, the optimum conditions slightly shifted to 0.078 J/mm^2^, obtained with a laser power of 20 W and a scan speed of 474.8 mm/s.
- The maximum tensile strength of the laser sintered PPSF is 9.259 MPa, obtained with an ED of 0.078 J/mm^2^.

## Figures and Tables

**Table 1:** Process conditions and tensile strength results of the multi-layered samples.

	Power (W)	Speed (mm/s)	Energy Density (J/mm^2^)	UTS (MPa)
A1	13	386.9	0.062	2.280
A2				1.666
A3				2.810
B1	20	595.0	0.062	6.561
B2				8.049
B3				7.102
C1	20	474.8	0.78	7.702
C2				8.994
C3				9.259

## Data Availability

The raw/processed data required to reproduce these findings cannot be shared at this time as the data also forms part of an ongoing study.
